# The Great Debate: CAR-T-Cell Therapy Versus Bispecific Antibodies in B-Cell Lymphoma

**DOI:** 10.3390/cancers18152513

**Published:** 2026-08-05

**Authors:** Massimo Martino, Violetta Marafioti, Martina Pitea, Gaetana Porto, Giorgia Policastro, Filippo Antonio Canale, Virginia Naso, Caterina Alati

**Affiliations:** Hematology and Stem Cell Transplantation and Cellular Therapies Unit (CTMO), Department of Hemato-Oncology and Radiotherapy, Grande Ospedale Metropolitano “Bianchi-Melacrino-Morelli”, 89133 Reggio Calabria, Italy; dr.massimomartino@gmail.com (M.M.); violetta.marafioti@ospedalerc.it (V.M.); porto.tania25@gmail.com (G.P.); giorgia.policastro@ospedalerc.it (G.P.); filippo.canale@ospedalerc.it (F.A.C.); virginia.naso@ospedalerc.it (V.N.); caterina.alati@ospedalerc.it (C.A.)

**Keywords:** CAR-T-cell therapy, bispecific antibodies, diffuse large B-cell lymphoma, axicabtagene ciloleucel, lisocabtagene maraleucel, epcoritamab, glofitamab, relapsed/refractory lymphoma, second-line therapy, third-line therapy

## Abstract

**Background:** The treatment paradigm for relapsed/refractory (R/R) large B-cell lymphoma (LBCL) has undergone significant change with the advent of CD19-directed chimeric antigen receptor T-cell (CAR-T) therapies and CD20 × CD3 bispecific antibodies (BsAbs). Although both approaches have shown high response rates in single-arm studies, the absence of prospective randomized head-to-head comparisons has resulted in true clinical equipoise. **Methods:** A narrative synthesis was conducted, incorporating pivotal and updated phase 2 and 3 trial data, real-world evidence, and published meta-analyses. **Results:** In the second-line setting, CAR-T therapy demonstrates superior event-free survival, progression-free survival, and overall survival compared to standard-of-care chemo-transplant regimens. In the third-line setting, a pooled meta-analysis indicates significantly higher complete response rates for CAR-T compared with BsAbs, as well as superior 12-month progression-free survival. BsAbs provide immediate availability, greater accessibility, more favorable neurotoxicity profiles, and are feasible for frail or elderly patients. Real-world data show that BsAb complete response rates are consistently lower than those observed in clinical trials, whereas CAR-T real-world effectiveness closely aligns with pivotal trial outcomes. Emerging phase 3 data on fixed-duration and monotherapy bispecific regimens suggest that a genuine, if less mature, curative fraction may also be achievable among BsAb-treated complete responders. **Conclusions:** CAR-T therapy remains the standard of care for fit, eligible patients with R/R LBCL in second- and third-line settings with curative intent, providing superior depth and durability of response and a growing potential for long-term cure. BsAbs constitute a critical therapeutic alternative for patients ineligible for CAR-T, those with rapidly progressive disease, frail or elderly individuals, and as bridging strategies. A patient-centered, scenario-specific clinical decision framework is recommended.

## Simple Summary

The therapeutic landscape for relapsed/refractory (R/R) large B-cell lymphoma (LBCL) has evolved with the introduction of CAR T-cell therapy and bispecific antibody therapy. The therapeutic landscape for R/R LBCL has evolved with the introduction of CAR T-cell therapy and bispecific antibody therapy. However, no randomized trials have directly compared these two modalities. For second-line treatment, CAR T-cell therapy demonstrates superior outcomes compared to standard chemotherapy, including improved event-free, progression-free, and overall survival. In the third-line setting, CAR T-cell therapy achieves higher complete response rates and superior 12-month progression-free survival relative to bispecific antibodies. Despite these advantages, bispecific antibodies are more readily available, less neurotoxic, and better suited for frail or elderly patients. In real-world settings, the effectiveness of bispecific antibodies is generally lower than observed in clinical trials, whereas CAR T-cell therapy outcomes remain consistent with trial data. Overall, CAR T-cell therapy remains the standard of care for eligible patients. Bispecific antibodies represent a critical alternative for individuals who are ineligible for CAR T-cell therapy, are frail, or require rapid initiation of therapy.

## 1. Introduction

Diffuse large B-cell lymphoma (DLBCL) and related aggressive B-cell lymphoma subtypes—collectively designated large B-cell lymphoma (LBCL) under the fifth edition of the World Health Organization Classification of Hematolymphoid Tumors (WHO-HAEM5) [1]—account for approximately 30–40% of all mature B-cell neoplasms [2], with an estimated incidence of 28,000 new cases per year in Europe [3]. Older nosologies grouped these entities under the broader umbrella term “non-Hodgkin lymphoma” (NHL); contemporary hematopathological classification favors entity-specific nomenclature, and this review retains the term NHL only where it reproduces the original wording of a cited historical dataset or registry category (Section 3) [1]. While approximately 60% of patients achieve durable remission with first-line rituximab-based immunochemotherapy (R-CHOP or equivalent) [4,5,6,7], a substantial proportion relapse or present with primary refractory disease [8]. Outcomes in this relapsed/refractory (R/R) setting have historically been poor: the international SCHOLAR-1 study reported a median overall survival of approximately 6 months among patients refractory to salvage chemotherapy [8]. For decades, the standard salvage strategy for fit patients consisted of platinum-based reinduction followed by high-dose chemotherapy and autologous stem cell transplantation (ASCT) [9,10], a paradigm that consolidated cure in only a minority of treated patients and left a substantial fraction of older or comorbid patients without a curative option.

Over the past decade, this therapeutic landscape has been fundamentally disrupted by two converging immunological revolutions. CD19-directed chimeric antigen receptor T-cell (CAR-T) therapies—axicabtagene ciloleucel (axi-cel), tisagenlecleucel (tisa-cel), and lisocabtagene maraleucel (liso-cel)—redirect autologous, genetically engineered T cells against CD19-expressing malignant B cells [11,12]. CD20 × CD3 bispecific antibodies (BsAbs)—epcoritamab and glofitamab—instead recruit and activate the patient’s own polyclonal T-cell repertoire against CD20-expressing tumor cells without requiring ex vivo cell engineering [11]. Both modalities have produced response rates unprecedented in the R/R setting and have been rapidly incorporated into international treatment guidelines, yet they differ profoundly in manufacturing logistics, toxicity spectrum, underlying T-cell biology, and patient eligibility criteria—differences that, as detailed in Section 2, preclude direct comparison through classical randomized controlled trial (RCT) design.

This review critically synthesizes evidence from pivotal trials, updated long-term follow-up data, real-world cohorts, and meta-analyses to address the central clinical question: in which patients, in which lines of therapy, and under which circumstances should CAR-T or BsAbs be the preferred strategy? Beyond efficacy and safety outcomes, we examine the underlying immunobiology of T-cell exhaustion and B-cell depletion that differentiates the two platforms (Section 2), the specific challenge of central nervous system (CNS) involvement (Section 8.3), and Italian real-world registry data that quantify this therapeutic transition in clinical practice (Section 3). A pragmatic, patient-centered clinical decision framework is proposed for the hematologist managing R/R LBCL (Section 9).

## 2. Mechanistic Background and Structural Differences

CAR-T-cell therapies are autologous, personalized cellular products engineered ex vivo through leukapheresis, retroviral/lentiviral transduction of T cells with a synthetic chimeric antigen receptor targeting CD19, and expansion in culture [12]. Following lymphodepleting conditioning (typically fludarabine and cyclophosphamide), the engineered product is infused as a single dose. The manufacturing process requires 2–6 weeks from leukapheresis to product release, during which disease must be controlled with bridging therapy. The CAR-T product then undergoes in vivo expansion, persistence, and cytotoxic activity via perforin/granzyme-mediated killing at the immunological synapse [13,14,15]. The fundamental advantage of CAR-T therapy is the potential for long-lived memory T cells capable of ongoing tumor surveillance, translating into durable remissions and, in a subset of patients, functional cure [16].

BsAbs, by contrast, are standardized, off-the-shelf molecules that simultaneously engage CD3 on T cells and CD20 on malignant B cells, forming an artificial immune synapse that activates endogenous T cells independent of MHC-peptide presentation or prior T-cell priming [17]. This mechanism enables rapid initiation—within days of the decision to treat—and administration in virtually any oncology setting, including centers not accredited by the Joint Accreditation Committee ISCT-Europe & EBMT (JACIE).The therapeutic activity of BsAbs, however, is dependent on continuous drug administration and the presence of adequate endogenous T-cell function, which may be compromised in heavily pre-treated patients [18]. The absence of a memory compartment means that, unlike CAR-T, tumor control is lost upon treatment discontinuation in most patients who do not achieve deep complete remission.

Four structural differences carry major clinical implications: (1) personalization versus standardization of the product; (2) single infusion versus repeated dosing; (3) long lead-time versus immediate availability; and (4) JACIE-accredited specialized center requirement versus broad accessibility in spoke centers. These differences make a classical head-to-head randomized comparison structurally problematic—the incompatible timelines of manufacturing (4–8 weeks) and immediate BsAb initiation preclude unified randomization windows—and unlikely to exist in the foreseeable future.

### Divergent T-Cell Biology: Exhaustion Versus Memory Formation

A growing body of evidence indicates that the two platforms engage distinct, and only partially overlapping, T-cell biology. CAR-T cells undergo a single, intense activation event driven by lymphodepleting chemotherapy and high-avidity synthetic-receptor engagement, followed by clonal expansion, contraction, and—in patients achieving durable remission—persistence of a long-lived central memory compartment capable of ongoing immunological surveillance [16]. BsAbs, in contrast, depend on repetitive and, in most protocols, indefinite engagement of the patient’s pre-existing polyclonal T-cell pool. Chronic antigen exposure under this dosing schedule drives progressive T-cell exhaustion, characterized by the upregulation of inhibitory receptors (PD-1, TIM-3, LAG-3), attenuated proliferative and cytotoxic capacity, and reduced cytokine production; treatment-free intervals have been shown to partially reverse this exhaustion phenotype in preclinical and early clinical studies [19]. This mechanistic distinction plausibly underlies the durable plateau observed with CAR-T versus the loss of disease control on BsAb discontinuation in most non-complete responders, and it helps explain why heavily pre-treated patients with exhausted or lymphopenic T-cell compartments may derive less benefit from either modality—a rationale that also underlies the use of BsAbs as an early bridging strategy, before T-cell fitness declines further.

**Figure 1:**
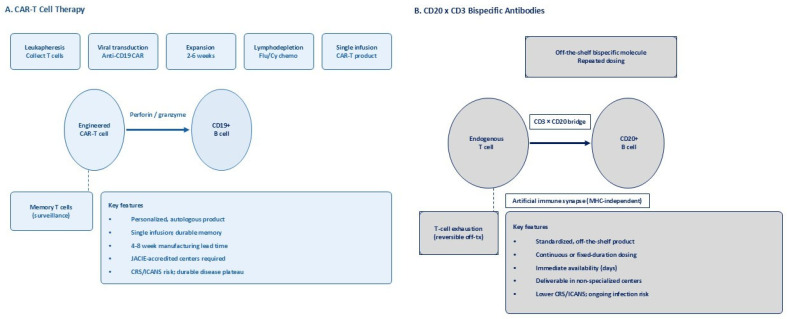
A schematic comparison of the mechanism of action, manufacturing logistics, and key clinical features of CAR-T-cell therapy (**A**) and CD20 × CD3 bispecific antibodies (**B**). Original figure prepared by the authors; Flu/Cy: fludarabine/cyclophosphamide.

The two platforms also differ in the kinetics and reversibility of B-cell depletion. CAR-T-associated B-cell aplasia results from on-target/off-tumor elimination of normal CD19^+^ B-cell precursors and can persist for months to years, tracking with CAR-T persistence and correlating with both durable tumor control and prolonged humoral immunosuppression, including impaired vaccine responses and hypogammaglobulinemia that requires immunoglobulin replacement in a subset of patients. BsAb-associated B-cell depletion is comparably profound during active dosing but is generally more rapidly reversible after treatment discontinuation, particularly with fixed-duration regimens such as glofitamab. This distinction has direct safety implications: the higher grade ≥3 infection rate observed with BsAbs when standardized per patient-month of exposure [20] likely reflects the combined burden of continuous T-cell engagement, sustained B-cell aplasia, and hypogammaglobulinemia accrued over a prolonged dosing interval, whereas CAR-T infection risk is concentrated in the early post-infusion period and subsequently declines as immune reconstitution proceeds (Figure 1).

## 3. The Changing Italian Landscape: GITMO Registry Data

Data from the Gruppo Italiano Trapianto di Midollo Osseo (GITMO) national registry [21] provide a striking real-world quantification of the therapeutic shift induced by novel immunological therapies in Italy (Table 1), complementing the trial and meta-analytic evidence discussed in Section 4, Section 5 and Section 6. Total autologous transplants peaked at 3578 in 2019 and have since declined steadily, reaching 3107 in 2025—a 9.6% reduction from 2023 and 2.6% from 2024. This secular decline is most dramatic in the NHL subtypes most directly affected by alternative T-cell-redirecting therapies.

Among LBCL patients specifically, autologous transplant volumes have declined by 47.2% from 360 (2020) to 190 (2025), mirroring precisely the period over which CAR-T therapy was introduced and expanded in Italy. This dramatic contraction is consistent with, and attributable to, the regulatory approval and clinical adoption of CAR-T therapy in both second-line (2L) high-risk DLBCL (axi-cel [22], liso-cel [23]; approved 2022 in Europe) and the continuing expansion of third-line and beyond (3L+) CAR-T utilization. Follicular lymphoma shows the largest relative decline (−64.8%), reflecting CAR-T adoption alongside novel fixed-duration regimens.

Conversely, CAR-T procedures in Italy have increased exponentially: from 43 in 2019 to 750 in 2025, representing a 14% year-on-year increase from 2024 (658 procedures). Lymphoma accounts for 74% of all CAR-T indications (*n* = 560/750 in 2025), with large B-cell lymphoma comprising 67% of the lymphoma subset (*n* = 378). In 2025, 50 certified Italian centers performed at least one CAR-T procedure; however, 23 centers performed fewer than 10 procedures per year, raising concerns about volume–outcome relationships and the maintenance of procedural expertise. The top performing centers (*n* = 3–4) account for a disproportionate share of national volume, highlighting persistent inequities in access.

## 4. Efficacy Data: CAR-T Therapies in 2L and 3L+

### 4.1. CAR-T in Second-Line DLBCL: Curative Intent

Two pivotal randomized phase 3 trials—ZUMA-7 and TRANSFORM—established CAR-T as the standard of care for transplant-eligible patients with primary refractory or early relapsing DLBCL (relapse within 12 months of first-line therapy).

In the ZUMA-7 trial (axi-cel vs. SoC, 2L) [22], axi-cel significantly improved event-free survival (mEFS: NR vs. 2.0 months, HR 0.40, *p* < 0.001) and was the first CAR-T product to demonstrate a statistically significant overall survival benefit versus the standard of care (HR 0.73, 95% CI 0.54–0.98, *p* = 0.03) at 47.2 months median follow-up. The estimated 4-year OS rate was 54.6% versus 46.0% for SoC. Importantly, 57% of SoC patients subsequently received 3L+ cellular immunotherapy off-protocol, suggesting that even with optimal post-progression salvage, the OS advantage of early CAR-T deployment is detectable. Quality-of-life recovery was significantly faster with axi-cel on both global health status and physical functioning subscales of the EORTC QLQ-C30 [24,25].

In TRANSFORM (liso-cel vs. SoC, 2L) [23], liso-cel demonstrated, at 49.4 months median follow-up, a 4-year progression-free survival (PFS) rate of 52.2% versus a historical SoC comparator (median PFS 6.2 months), with median PFS not reached in the liso-cel arm—suggesting a true plateau consistent with durable cure in a subset of patients.

Real-world evidence from the US Center for International Blood and Marrow Transplant Research (CIBMTR) registry (n = 446, median follow-up 12 months) confirmed trial outcomes in a broader patient population [26]: the 12-month EFS was 53% overall (58% ZUMA-7-eligible; 48% ZUMA-7-ineligible), and 12-month OS was 62% in trial-ineligible and 80% in trial-eligible patients. Critically, approximately half of real-world patients would have been excluded from ZUMA-7, yet outcomes remained clinically meaningful, supporting the general applicability of 2L CAR-T. A combined analysis of axi-cel across ZUMA-7 (transplant-eligible) and ALYCANTE (transplant-ineligible) reinforces this conclusion, demonstrating virtually identical 24-month efficacy outcomes irrespective of ASCT eligibility (EFS 45.2% and 44.6%, respectively; OS 62.8% and 70.8%) [27].

### 4.2. CAR-T in Third and Later Lines: Evidence for Functional Cure

Long-term follow-up of ZUMA-1 (axi-cel, 3L+, n = 101; mFU 63.1 months) has provided compelling evidence for the curative potential of CAR-T in later-line DLBCL25: 5-year OS was 42.6%, 5-year LREFS was 33.5%, and approximately four of five patients alive at 5 years who had previously responded are estimated to be potentially cured—defined operationally by a plateau on LREFS curves beyond 24 months. The landmark duration of complete response (DoCR) analysis showed a median DoCR of 34.7 months for CRs achieved at week 4, and not reached for CRs achieved after week 4, with a median duration of CR of 62.2 months. Real-world data for liso-cel (n = 1116) showed a 12-month OS of 67.6% overall, with 2L LBCL patients achieving a 12-month OS of 65.1% and median PFS not reached [28].

## 5. Efficacy Data: Bispecific Antibodies in 3L+ and 2L

### 5.1. Monotherapy BsAbs in 3L+

Epcoritamab achieved an ORR of 63% and a CR rate of 39% in patients with R/R LBCL and ≥2 prior lines (*n* = 157; mFU 30.6 months) [29]. Time to response was rapid (median 40 days), which represents a critical practical advantage. However, at 30.6 months follow-up, 85% of patients had discontinued treatment—primarily due to progressive disease (*n* = 90) or Adverse events (*n* = 23). The subcutaneous administration schedule (weekly × 12, biweekly × 24 weeks, then monthly) requires step-up dosing for CRS management but is largely compatible with outpatient delivery.

Glofitamab demonstrated ORR 52%, CR 40% in a similar 3L+ population (n = 155; mFU 32 months), with the critical advantage of a fixed treatment duration (12 cycles, approximately 8.5 months) [30]. However, only one in four patients completed the full course, largely due to disease progression. Time to full dose was 21 days; time to best response 42 days.

Multiple real-world cohorts consistently report lower CR rates than pivotal trials: for glofitamab, real-world CR rates range 25–32% [31,32,33,34]; for epcoritamab, 23–31% [33,34]. This systematic dilution in real-world settings reflects the inclusion of patients with more adverse prognostic features, greater prior treatment burden, and performance status limitations. Nonetheless, longer follow-up increasingly supports genuine curative potential for a subset of BsAb complete responders, particularly with fixed-duration regimens. Among glofitamab-treated patients who achieve CR, an estimated 79% remain in complete response two years after treatment discontinuation, suggesting an indefinite treatment-free interval that parallels the CAR-T plateau [30]. This picture is further reinforced by EPCORE DLBCL-1, the first randomized, phase 3 evaluation of a CD3 × CD20 BsAb given as monotherapy, presented at the European Hematology Association (EHA) 2026 Congress: epcoritamab significantly improved PFS over investigator’s-choice chemoimmunotherapy in second-line R/R LBCL (24-month PFS 30% vs. 13%; HR 0.74, 95% CI 0.60–0.92, *p* = 0.0059), with a markedly longer median duration of CR (not reached vs. 10.8 months) and higher CR rate (38% vs. 26%) [35]. The co-primary OS endpoint was not met in the primary analysis (HR 0.96, 95% CI 0.77–1.20); investigators attributed this to concentrated enrollment during the COVID-19 pandemic and to imbalanced access to effective post-progression therapy between arms (31% vs. 6%), with a posthoc adjustment for both factors shifting the OS HR to 0.76 (95% CI 0.59–0.99) [35]. These emerging data temper, without overturning, the conclusion that durable disease control with BsAbs remains, at present, less consistently demonstrated than with CAR-T; they support cautious optimism that a genuine curative fraction exists among BsAb-treated complete responders, and they warrant close monitoring as longer-term follow-up matures.

### 5.2. Glofitamab-GemOx in Transplant-Ineligible 2L Patients (STARGLO)

STARGLO randomized 274 transplant-ineligible R/R DLBCL patients 2:1 to Glofit-GemOx versus R-GemOx for up to 12 cycles [36,37]. The trial reported superior OS (HR 0.59, 95% CI 0.40–0.89) and superior ORR/CR for the glofitamab arm overall. However, the results are critically complicated by profound regional heterogeneity. FDA analysis revealed that the survival benefit was entirely driven by the Asian subpopulation (n = 131; HR 0.39, 95% CI 0.25–0.63), while no benefit was detectable in non-Asian patients (n = 143; HR 1.06, 95% CI 0.61–1.84). In the US subgroup, the HR for OS was 2.62 (95% CI 0.56–12.34). Three explanatory factors were identified: (1) Asian patients were substantially younger (median 62 vs. 71 years) and 65% were ASCT-ineligible solely because they refused transplantation—not due to comorbidities—selecting a more fit population; (2) the R-GemOx control arm was intrinsically weaker in Asia due to fewer post-progression treatment options; and (3) prognostic imbalances in the non-Asian experimental arm (higher proportion of primary refractory disease in the glofitamab arm). No pharmacokinetic explanation for regional differences has been identified. In May 2025, an FDA Oncologic Drugs Advisory Committee voted 8-1 that the STARGLO results were not applicable to the US patient population [38].

## 6. Comparative Evidence: Meta-Analyses and Cross-Trial Synthesis

The fundamental challenge in comparing CAR-T and BsAbs is structural: different approved indications (2L+ vs. ≥3L), incompatible timelines (4–8-week manufacturing vs. immediate BsAb initiation), and divergent infrastructure requirements (JACIE-accredited centers vs. standard oncology facilities) render classical head-to-head RCT design unfeasible—and unlikely to materialize. The only comparative evidence derives from single-arm meta-analyses and cross-trial syntheses (Table 2).

The largest and most rigorous of these syntheses, conducted by Kim and colleagues [39], pooled 16 trials enrolling 1347 patients with R/R DLBCL treated in ≥3L—six BsAb trials (n = 593) and 10 CAR-T trials (n = 713). The primary finding was a significantly higher pooled CR proportion for CAR-T (0.51, 95% CI 0.46–0.56) versus BsAbs (0.36, 0.29–0.43; subgroup difference *p* < 0.01). This advantage persisted in the pre-specified sensitivity analysis restricting BsAb patients to CAR-T-naive recipients (CR 0.37, 0.32–0.43). Twelve-month PFS was also significantly higher for CAR-T (0.45 vs. 0.37, *p* = 0.02). The advantage of CAR-T was confirmed by multivariate meta-regression adjusting for double-hit lymphoma proportion. Methodological limitations include the heterogeneity of patient populations, variable follow-up duration across trials, and the confound that a majority of BsAb trial patients had prior to CAR-T exposure.

On safety, the same meta-analysis demonstrated that CAR-T carries a significantly higher risk of severe (grade ≥3) CRS (8% vs. 2%), severe immune effector cell-associated neurotoxicity syndrome (ICANS; 11% vs. 1%), and severe infections (17% vs. 10%). However, a nuanced reappraisal is required for the infection comparison: van Besien and colleagues (15 CAR-T + 10 BsAb studies; n = 3202 patients) demonstrated that when infection rates are standardized per patient-month—accounting for the continuous and indefinite nature of BsAb dosing versus the single-episode CAR-T infusion—bispecific antibodies carry a significantly higher cumulative infection burden, particularly for grade ≥3 infections [20]. This finding, consistent with the B-cell depletion kinetics discussed in Section Divergent T-cell Biology: Exhaustion Versus Memory Formation, has profound implications for elderly or immunocompromised patients who may be on BsAb therapy for months to years.

## 7. Summary of Key Pivotal Trials

The pivotal trials discussed individually in Section 4 and Section 5 are summarized together below for direct comparison, alongside their key efficacy findings and safety signals (Table 3).

## 8. Patient-Centered Clinical Decision Framework

Given the structural impossibility of a head-to-head RCT and the complexity of the available evidence, clinical decisions must be individualized according to patient fitness, disease characteristics, logistical constraints, and institutional capabilities—including performance status as measured by the Eastern Cooperative Oncology Group (ECOG) scale. The framework below (Table 4) is grounded in EHA Clinical Practice Guidelines [41], NCCN v6.2026 recommendations [42], and the evidence synthesized in this review.

### 8.1. The Case for CAR-T as the Preferred Strategy in Eligible Patients

The evidence overwhelmingly supports CAR-T as the preferred strategy for patients who are medically fit, have adequate functional reserves for the treatment process (including lymphodepletion and potential ICU-level CRS/ICANS management), have access to a JACIE-certified center, and can tolerate a 4–8 week manufacturing waiting period with adequate bridging. The rationale is threefold:

- Depth of response: pooled CR rates 51% (CAR-T) versus 36% (BsAbs) at 3L+, with superior rates in 2L (65% for liso-cel/axi-cel) [39].
- Durability: CAR-T demonstrates a well-established plateau on PFS/OS curves beyond 24–36 months in the majority of trials to date [40,43,44]. Emerging fixed-duration and monotherapy BsAb data (Section 5.1) [30,35] suggest that a comparable, if less mature, plateau may be achievable in patients who achieve CR, narrowing—without yet closing—this historical gap.
- Curative intent: ZUMA-1 5-year data25 and ZUMA-7 4-year OS data19 document that CAR-T therapy can achieve durable, potentially lifelong remission—a threshold that BsAbs, at the current evidence level, cannot demonstrate. The quality-of-life advantage of CAR-T versus SoC chemotherapy [24,25] (faster recovery on EORTC QLQ-C30 global health and physical functioning) is an additional argument for early CAR-T over repeated cycles of chemotherapy.

### 8.2. The Case for Bispecific Antibodies: When CAR-T Is Not the Answer

BsAbs are not an inferior alternative—they are the appropriate first choice in several clinically important scenarios:

- Rapidly progressive disease: Patients with fast-growing LBCL who cannot safely await 4–8 weeks of manufacturing benefit from immediate BsAb initiation, which achieves responses within 4–6 weeks. BsAbs may also serve as effective bridges to subsequent CAR-T, with reassuring data showing that prior BsAb exposure does not materially impair CD19 expression or CAR-T efficacy (CD19 downregulation impacts <5% of cases clinically) [45,46].
- Elderly or frail patients (age ≥70yr, ECOG PS ≥2, cardiac/neurologic/cognitive comorbidities): The ICANS G ≥ 3 rate of 10–32% with CAR-T may be unacceptable in patients with pre-existing CNS vulnerability. BsAbs carry <2% ICANS G ≥ 3 and are largely manageable in outpatient or spoke-center settings.
- Post-CAR-T relapse (3L+ after cellular therapy failure): BsAbs demonstrate meaningful activity (ORR ~30–40%) without complete cross-resistance, representing the primary salvage option in this setting [47,48].
- Geographical or institutional inaccessibility: In total, 23 of 50 Italian CAR-T centers perform fewer than 10 procedures per year [21], raising expertise concerns. BsAbs can be administered in any standard oncology facility, directly addressing the equity gap.

The infection risk reframing by van Besien et al. [20] requires attention: while per-event CRS/ICANS rates are lower with BsAbs, the cumulative infection burden per patient-month during prolonged BsAb administration may exceed that of the single CAR-T infusion episode, particularly in immunocompromised or elderly patients.

### 8.3. Management of Patients with CNS Involvement

Secondary CNS involvement by aggressive LBCL carries a historically dismal prognosis and represents a distinct management challenge for both platforms. Patients with active CNS disease were largely excluded from the pivotal CAR-T and BsAb registration trials discussed in Section 4 and Section 5, so evidence in this setting derives chiefly from retrospective and registry cohorts and dedicated expert reviews [49]. A EBMT/GoCART coalition analysis of 100 patients with CNS-involved LBCL treated with CD19 CAR-T reported meaningful response rates, although concern for ICANS—a neurotoxicity syndrome that shares clinical features with CNS lymphoma progression and can complicate diagnostic interpretation—has limited broader trial enrollment in this population [50]. BsAbs were similarly excluded from pivotal trials owing to uncertainty regarding blood–brain barrier penetration and theoretical concerns about exacerbating neurotoxicity; however, glofitamab has been detected at pharmacologically active concentrations in cerebrospinal fluid, and a multi-center retrospective series of CD3 × CD20 BsAb therapy in primary and secondary CNS lymphoma reported encouraging response rates (50–80%) in this previously excluded population [51]. Until prospective data become available, current expert guidance [49] favors individualized, multidisciplinary decision-making—incorporating intrathecal or high-dose methotrexate-based induction, careful neurologic monitoring during T-cell-redirecting therapy, and enrollment in dedicated clinical trials whenever feasible—over a uniform algorithm for either modality.

## 9. Future Directions and Conclusions

### 9.1. Future Directions and Open Questions: Several Critical Unresolved Questions Will Shape the Next Phase of This Debate

- Earlier-line deployment: Multiple trials are exploring axi-cel and liso-cel in 1L high-risk DLBCL [52], and BsAbs in 1L/2L combination regimens [53], including the EPCORE DLBCL-1 monotherapy platform now being considered for regulatory discussion in 2L (Section 5.1) [35]. Front-line results may further reshape the role of 2L cellular therapy.
- Combination strategies: BsAb + CAR-T sequential or concurrent combinations, BsAb + lenalidomide, BsAb + checkpoint inhibitors, and next-generation dual-targeting CAR-T constructs are under investigation.
- Allogeneic CAR-T: Off-the-shelf, donor-derived CAR-T products could eliminate the manufacturing lead-time disadvantage, potentially combining the logistical advantages of BsAbs with the durability advantages of cellular therapy [54].
- Predictive biomarkers: Identification of patients most likely to achieve durable remission with CAR-T (ctDNA kinetics, tumor microenvironment characteristics, T-cell fitness metrics, CD19 expression dynamics) versus those who may respond adequately to BsAbs remains an urgent research priority.
- Access equity: The geographic concentration of CAR-T expertise in high-volume centers and the economic burden of CAR-T manufacturing must be addressed through hub-and-spoke organizational models, telemedicine-supported follow-up, and health technology assessment frameworks.
- Prospective real-world registries: Given the structural impossibility of a head-to-head RCT, prospective, multi-center observational registries with pre-defined comparative endpoints and standardized data collection represent the most feasible path to comparative effectiveness evidence.
- CNS-directed evidence: Dedicated prospective studies of both CAR-T and BsAbs in patients with active or prior CNS involvement are urgently needed to move beyond the retrospective, registry-based evidence summarized in Section 8.3.
- OS as a regulatory bar for BsAb monotherapy: Evolving FDA guidance increasingly frames overall survival as both an efficacy and safety endpoint for novel immunotherapies; how regulators weigh PFS gains against unmet OS endpoints, as in EPCORE DLBCL-1, will shape the pace at which BsAb monotherapy moves into earlier treatment lines [35].

### 9.2. Conclusions

The debate between CAR-T and bispecific antibodies in aggressive B-cell lymphoma does not resolve into a simple hierarchy of superiority. Rather, it defines a complementary ecosystem of immunological therapies, each occupying a distinct and essential niche in the treatment algorithm.

CAR-T-cell therapy—with its demonstrated curative potential, superior response depth, and durable plateau on survival curves—remains the standard of care for medically fit, eligible patients in 2L high-risk DLBCL and represents the preferred strategy in 3L+ whenever feasible. The sustained 4-year OS benefit of axi-cel and the plateau PFS of liso-cel in the 2L randomized setting have established cellular therapy as a cornerstone of potentially curative treatment.

Bispecific antibodies—with their immediate availability, outpatient feasibility, favorable neurotoxicity profile, and accessibility in non-specialized centers—are the treatment of choice for elderly or frail patients, rapidly progressive disease, post-CAR-T relapse, and geographically underserved populations. Maturing phase 3 monotherapy and fixed-duration data (Section 5.1) [30,35] indicate that a meaningful curative fraction may also be achievable among BsAb complete responders, even though this evidence remains less mature than for CAR-T. The STARGLO trial, while positive overall, requires cautious interpretation in the Western patient population given its profound regional heterogeneity and the FDA ODAC assessment.Italian GITMO data provide a real-world barometer of this therapeutic transition: a 47.2% collapse in LBCL autologous transplants alongside a 14% year-on-year growth in CAR-T procedures reflects a genuine paradigm shift in clinical practice. The challenge for the next decade is not to choose between these modalities, but to sequence, combine, and allocate them optimally—guided by patient biology, clinical context, and a commitment to equitable access.

## Figures and Tables

**Table 1:** Italian GITMO registry: autologous transplant trends by lymphoma subtype (2020 vs. 2025). Source: GITMO national registry [21]. Percentage change relative to 2020 baseline. LBCL: large B-cell lymphoma; FL: follicular lymphoma; MCL: mantle cell lymphoma; Auto-SCT: autologous stem cell transplant. * Corresponds to the registry category historically termed “non-Hodgkin lymphoma (NHL)” [1].

Lymphoma Subtype	Auto-SCT 2020	Auto-SCT 2025	Changed Percentage (%)
All B-cell lymphoma subtypes (total) *	768	496	−35.4%
Large B-cell lymphoma (LBCL)	360	190	−47.2%
Follicular lymphoma (FL)	108	38	−64.8%
Mantle cell lymphoma (MCL)	158	131	−17.1%
Others	142	137	−3.5%

**Table 2:** Comparative efficacy and safety: CAR-T vs. bispecific antibodies (meta-analysis summary). Data derived from Kim et al. [39] (Blood 2024; 144:629–638) and van Besien et al. [20] (Blood Advances 2025; 9:6063–6075). CAR-T: chimeric antigen receptor T-cell therapy; BsAbs: bispecific antibodies; CR: complete response; PFS: progression-free survival; CRS: cytokine release syndrome; ICANS: immune effector cell-associated neurotoxicity syndrome. * Per patient-month standardization reveals a higher cumulative infection burden with BsAbs due to continuous dosing.

Outcome	CAR-T (Pooled)	BsAbs (Pooled)	*p*-Value	Interpretation
CR Rate	0.51 (0.46–0.56)	0.36 (0.29–0.43)	<0.01	CAR-T superior
PFS at 12 months	~40–45%	~30–35%	0.02	CAR-T superior
Durable response at 2 year	~30–40%	~20–30%	—	Plateau observed with CAR-T
CRS grade ≥3	8% (0.08)	2% (0.02)	0.03	BsAbs safer
ICANS grade ≥3	11%	1%	<0.01	BsAbs safer
Grade ≥3 infection (per patient-month)	0.17 (0.11–0.22)	0.10 (0.03–0.16)	<0.01 *	BsAbs higher risk (cumulative/continuous dosing)

**Table 3:** Key pivotal trials: CAR-T and bispecific antibodies in R/R LBCL.

Trial	Agent	Line	N	ORR (%)	CR (%)	Median PFS	Key Finding	Ref.
**CAR-T-Cell Therapies**								
**ZUMA-7**	Axi-cel	2L (HR)	359	83	65	NR vs. 2.0 month	First CAR-T with OS benefit (*p* = 0.03); 4 year OS 54.6%.	[22]
**TRANSFORM**	Liso-cel	2L (HR)	184	86	66	NR vs. 2.4 month	4 year PFS 52.2% (liso-cel arm). Median PFS not reached at 49mo follow-up.	[23]
**ZUMA-1**	Axi-cel	3L+	101	83	58	—	5 year OS 42.6%; ~4/5 responders alive at 5 year potentially cured.	[40]
**TRANSCEND NHL 001**	Liso-cel	3L+	269	73	53	12 month (median)	Favorable safety: CRS G ≥ 3 only 4%, ICANS G ≥ 310%.	[13]
**Bispecific Antibodies**								
**EPCORE NHL-1**	Epcoritamab	3L+	157	63	39	4.4 month	85% discontinued at 30.6mo mFU; SC administration; 24 h hospitalization only for CRS prophylaxis.	[29]
**NP30179 (Glofitamab)**	Glofitamab	3L+	155	52	40	4.9 month	Fixed 12-cycle duration; time-limited therapy. Only 1/4 patients completed treatment.	[30]
**STARGLO**	Glofit-GemOx	2L+ (ASCT-ineligible)	274	68 vs. 42	59 vs. 25	13.8 vs. 3.6 month	Significant regional variation: benefit driven by Asian subgroup. FDA ODAC voted 8-1 results not applicable to US population.	[37]
EPCORE DLBCL-1	Epcoritamab	2L	483	51 vs. 48	38 vs. 26	24-month PFS 30% vs. 13%	First randomized phase 3 CD3 × CD20 BsAb monotherapy to improve PFS vs. chemoimmunotherapy; OS endpoint not met in primary analysis.	[35]

Axi-cel: axicabtagene ciloleucel; liso-cel: lisocabtagene maraleucel; Glofit-GemOx: glofitamab + gemcitabine–oxaliplatin; HR: high-risk (primary refractory or relapse <12 months); NR: not reached; SoC: standard of care (chemo-mobilization + high-dose chemotherapy [HDCT] + ASCT); ORR: overall response rate; CR: complete response; PFS: progression-free survival; OS: overall survival; SC: subcutaneous. Reference numbers are listed in the rightmost column.

**Table 4:** Clinical decision framework: CAR-T versus bispecific antibodies in R/R LBCL.

Clinical Scenario	CAR-T	BsAb	Rationale
Fit patient, ≥2 L, JACIE center, stable disease	PREFERRED axi-cel or liso-cel	Alternative if ineligible	CR and OS superiority documented. Sustained plateau observed. Acceptable bridging window. EHA guidelines recommend axi-cel/liso-cel [I,A].
High-risk early relapse (<12 month) or R-CHOP-refractory	PREFERRED liso-cel/axi-cel 2L	Only if not CAR-T eligible	TRANSFORM: 1yr EFS 44% vs. 24% SoC. ZUMA-7: OS benefit *p* = 0.03. Both approved in 2L DLBCL by EMA/FDA.
Rapidly progressive disease (bridging ≥4–8 weeks unfeasible)	Not feasible (lead-time 4–8 weak)	FIRST CHOICE Rapid response C1-2	BsAbs achieve responses within 4–6 weeks. Can serve as bridge to CAR-T once disease controlled.
Age ≥70 year or cardiac/neurologic/cognitive comorbidities	ICANS 10–32% unacceptable risk	PREFERRED ICAN <2%, outpatient	Favorable toxicity profile. No hospitalization required (except step-up CRS prophylaxis). Caregiver burden lower.
ECOG PS ≥2 or severe frailty	Not eligible (standard criteria)	FIRST CHOICE (EPCORE, NP30179 included PS2)	Pivotal BsAb trials enrolled ECOG PS2. Step-up dosing feasible in outpatient/spoke centers.
3L+ after CAR-T failure	Already performed or not repeatable	FIRST CHOICE ORR ~30–40%	No complete cross-resistance. Activity preserved post-CD19 CAR-T. No alternative curative option in most.
Bridge to CAR-T during manufacturing (4–8 week wait)	Awaiting	OPTION as bridge	Reassuring data: CD19 downregulation <5% clinical impact. Meta-analysis confirms no impairment of subsequent CAR-T efficacy.
Double-hit/triple-hit lymphoma (MYC + BCL2/BCL6)	PREFERRED CR rate documented	Active but limited DHL data	Kim 2024 [39] meta-regression: CAR-T advantage persists in DHL even after multivariate adjustment for DHL proportion.
Active or prior CNS involvement	Individualized; limited trial data (Section 8.3)	Individualized; limited trial data (Section 8.3)	Both platforms largely excluded active CNS disease from pivotal trials; multidisciplinary, registry-informed decision-making required.
No access to JACIE center/geographically remote area	Not practically available	ONLY FEASIBLE OPTION	BsAbs deliverable in spoke centers. Critical equity consideration. Reduces geographic treatment gap.

JACIE: Joint Accreditation Committee ISCT-Europe & EBMT; ICANS: immune effector cell-associated neurotoxicity syndrome; EFS: event-free survival; DHL: double-hit lymphoma; BsAb: bispecific antibody; PS: performance status; ECOG: Eastern Cooperative Oncology Group; CNS: central nervous system.

## Data Availability

No new data were created or analyzed in this study. Data sharing is not applicable to this article, as all data discussed are available within the cited publications.
